# Effects of Experimental Hyperthyroidism on Collagen IV and Laminin-α5 Gene Expression in Balb/C Mouse Seminiferous Tubules

**DOI:** 10.31661/gmj.v8i0.1369

**Published:** 2019-11-09

**Authors:** Saeed Sadeghi, Mahdi Jalali, Mohammad Reza Nikravesh, Mojtaba Sankian

**Affiliations:** ^1^Department of Anatomy and Cell Biology, School of Medicine, Mashhad University of Medical Sciences, Mashhad, Iran; ^2^Immunology Research Center, School of Medicine, Mashhad University of Medical Sciences, Mashhad, Iran

**Keywords:** Collagen Type IV, Laminin, Gene Expression, Hyperthyroidism

## Abstract

**Background:**

Hyperthyroidism is one of the disorders of the thyroid gland, an organ that controls the cellular and molecular behaviors of the seminiferous tubule basement membrane (BM), and ultimately, influences its evolutionary process. We aimed to investigate the effects of hyperthyroidism on immunohistochemical characteristics and gene expression levels of collagen IV and laminin-α5 in seminiferous tubules BM of Balb/C mice.

**Materials and Methods:**

Twenty male Balb/C mice were divided into experimental and control groups. The experimental group received 500 mg/l of levothyroxine (L-thyroxine) diluted in drinking water for two months to inducing hyperthyroidism, which was confirmed by radioimmunoassay. At the end of the study, the mice were sacrificed, and their testes were extracted for immunohistochemistry and real-time polymerase chain reaction assays.

**Results:**

Although a weak reaction was observed in the experimental specimens, no significant enhancement was noted in color intensity of type IV collagen in the seminiferous tubules BM of the experimental group as compared to the control group (P>0.05). Collagen IV gene expression results in the experimental group were not significantly different from the controls (P>0.05). Thus, there was a significant increase in laminin α5 gene expression compared to the control group (P=0.016).

**Conclusion:**

Considering the key role of collagen IV and laminin-α_5_ in the seminiferous tubule BM in the testes, the results of this study indicated that hyperthyroidism has important effects on both structures and functions of these two components.

## Introduction

Thyroid hormones (THs) have cardinal effects on the growth and development of nearly all tissues and organs, especially the testes and process of spermatogenesis [1]. This hormone with its receptors can affect a wide range of genes. Most genes are up-regulated, and some may be down-regulated by THs and produce complex signaling [2]. Testicular tissue includes a large number of seminiferous tubules, which are firmly maintained by connective tissues. The main structure of the basement membrane (BM) of these tubules is formed by elastic fibers and myoid cells. Also, Sertoli cells in the BM form a blood-testis barrier [3]. Hyperthyroidism is accompanied by an increase in both T4 and T3 hormones. High levels of THs induce damage to testicular tissue by reducing its mass, the number of Sertoli cells, and sperm motility, thus, decrease the concentration of testosterone in blood plasma. TH nuclear receptors TRα and TRβ are located on Sertoli cells [4, 5]. Extracellular matrix, which plays an important role in the development of cytoskeletons, is a complex structure made of protein molecules, glycoprotein, fibronectin, laminin, collagen, and other polysaccharides. Fibronectin is located under the peritubular myoid cells in the BM of testes and plays a role in cell adhesion, blood coagulation, and metastasis [6, 7]. BM is one of the most specialized parts of the extracellular matrix. This structure includes two main proteins of laminin and collagen. Laminin is produced by fibroblasts and epithelial cells; laminin is a flexible protein with three long chain polypeptides and disulfide bonds in the form of a cross shape, which plays vital roles in the mesenchymal part of BM in the fetal and postnatal periods of Balb/C mice [8, 9]. Each laminin molecule consists of three subunits of α (α_1_-α_5_), β (β_1_-β_4_), and ɣ (ɣ_1_-ɣ_3_) chains, which are fundamental factors in the polarization and morphogenesis of BM in all tissues [10, 11]. On the other hand, 30% of the body^’^s proteins are collagens. Collagen IV is abundant in the lamina, a dense layer in BM that appears during the growth and development of mouse embryo. It seems that laminin and collagen IV stimulate the process of differentiation [12-14]. Collagen IV creates a solid mechanical scaffold as a ligand for integrins and other cellular receptors; it produces signaling pathways with growth factors such as bone morphogenetic proteins (BMPs) [15, 16]. Due to the close association between thyroid glands and genital secretions, especially the testicles, this study was conducted to investigate the increase in thyroid gland hormones in hyperthyroidism, its effects on the two main components of BM, namely collagen IV and laminin, intercellular matrix in testicular tissue, and changes in the results compared with the control sample. Herein, we aimed to determine the effect of hyperthyroidism on the gene expression of collagen IV and laminin-α5 and other changes in seminiferous tubules of Balb/C mice.

## Materials and Methods

### Animals

In this study, adult male Balb/C mice weighing 25-30 g were obtained from the animal house of Faculty of Medicine, Mashhad University of Medical Sciences, Iran, and housed in standard conditions (12:12 light: dark cycle at a temperature of 22-24°C and humidity of 50-55%). During the experiments, the mice were allowed free access to food and water. The mice were used in accordance with the standards of Animal Research Ethics Committee of Mashhad University of Medical Sciences, Iran (code of ethics: IR.MUMS.fm.REC.1394.306).

### Group and Design

After adapting to the new environment, the mice were randomly divided into two groups of control (without any interventions) and experimental (received 500 mg/l of levothyroxine [L-thyroxine] as a solution for about two months) [17].

### Assessment

Radioimmunoassay (RIA) was performed on the blood serums for confirmation of hyperthyroidism. After the treatment, all the mice were sacrificed, and the right testes were fixed in formalin. Based on the standard histological methods, the samples were molded in paraffin, and the blocks were cut at a thickness of 5 μm for immunohistochemical and real-time polymerase chain reaction (RT-PCR) studies of collagen IV and laminin-α5.

### Immunohistochemistry

To perform immunohistochemistry assay, the samples were first placed in xylene (Merck, Germany) for deparaffinization and in downgraded alcohol (Sina, Iran) for dehydration. After washing with distilled water, the specimens were washed three times for 15 min with phosphate buffer saline (PBS; pH=7.4). For removing antigens, the samples were placed in PBS and ethylenediaminetetraacetic acid (EDTA, Sigma, USA) solution in a warm water bath at 100°C for 30 min. Then, they were washed with PBS for 15 min. To deactivate internal peroxidase, the samples were immersed in methanol/water solution for 20s and washed with PBS for 15 min. To disable non-specific antigens, we used blocker solution combined with 1% bovine serum albumin (BSA1%, Sigma, USA) and serum goat 10% (Abcam, USA) in PBS for 30 min. After that, the specimens were incubated with primary antibodies of collagen IV (No:19808, Abcam, USA) and laminin-α5 (No:75344, Abcam, USA) at a concentration of 1:100 for 24 h at 4°C. After washing with PBS for 15 min, the samples were incubated with goat polyclonal conjugated with horseradish peroxidase IgG (No:7061, Serotec, USA) at a concentration of 1:400 for 2 h at 37°C and then washed with PBS for 15 min. Afterward, the samples were exposed to a 0.03% solution of 3,3’-diaminobenzidine (Sigma, USA) containing 0.01% H_2_O_2_ substrate for 15 min. Localized collagen and laminin proteins should be brown. After washing with 1.5 g of water and distilled water, the samples were stained with hematoxylin for background color. The samples were placed in ascending concentrations, and then Xylene washes were used to increase the transparency of the samples. The samples were placed on slides, and the sections were examined by optical microscopy (BX51, Olympus, Japan) connected to a camera with a magnification of 100x. Two researchers in double-blind form reviewed the images. The color intensity of the samples in different groups was categorized semi-quantitatively (Table-1) [10, 15]. According to Table-1, based on the immunohistochemical staining intensity, the samples were classified according to anti-collagen or anti-laminin antibodies from bright to dark brown.

### Collagen IV and Laminin-α5 Genes mRNA Expression

Total RNA of testis samples was isolated by Total RNA Purification Kit (Parstous Biotechnology, Iran) according to the manufacturer’s instructions. Integrity and purity of the extracted RNA were evaluated by electrophoresis on Green Viewer (Parstous Biotechnology, Iran) stained 1.5% agarose gel and visualized by UV transilluminator. The first-strand cDNA of the extracted mRNA was synthesized by Easy cDNA Synthesis kit (Parstous Biotechnology, Iran) in the presence of oligo (dT)_16_ primers (Parstous Biotechnology, Iran). mRNA transcripts of laminin-α5 and collagen IV were examined by quantitative SYBR Green real-time PCR kit (Parstous Biotechnology, Iran) and employing the mouse glyceraldehyde-3-phosphate dehydrogenase (GAPDH) gene as a housekeeping gene. Primes are listed in Table-2. Each reaction (10 µl) contained 5 µl of SYBR 2X Master Mix and 0.5 µM of each specific primer. Amplification was performed with an initial denaturation at 95^◦^C for 10 min, followed by 45 cycles of amplification (94^◦^C for 20 s and 60^◦^C for 30 s) using a Rotor-Gene Q (Qiagen, Germany).Reaction mixtures were heated to 72^◦^C for 3 min and then ramped to 99^◦^C to analyze melting curves.To evaluate gene expression, the cycle threshold (Ct) for collagen and laminin genes was normalized to that of the housekeeping gene (GAPDH). The relative quantification value of the target is expressed as 2−ΔΔCt, where ΔCt=Ct (specific gene)–Ct (GAPDH) and ΔΔCt=ΔCt (sample)– ΔCt (calibrator). All the data regarding the drugs used in this study are shown in Table-2.

### Statistical Analysis

To analyze the data, the Mann-Whitney U test and t-test were applied for immunohistochemical evaluations in SPSS version 16 (SPSS Inc., Chicago, Illinois, USA). A P-value of less than 0.05 was considered as statistical significant.

## Results

**Figure 1:**
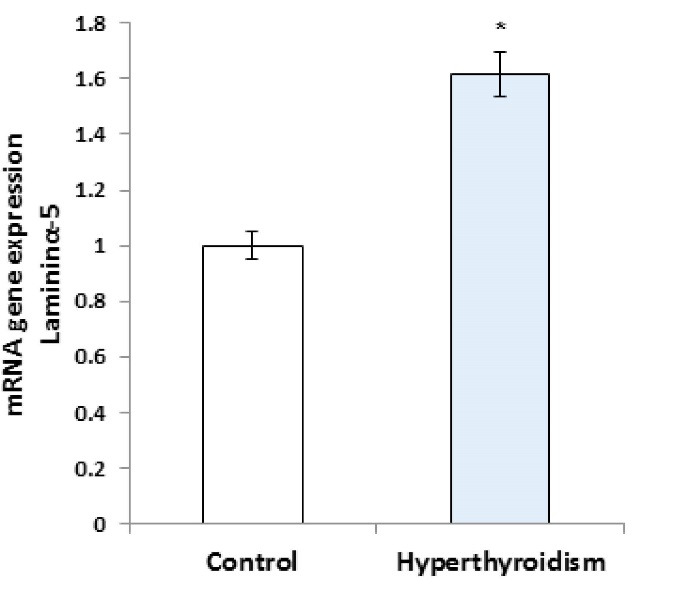
Laminin mRNA gene expression in the basement membrane of seminiferous tubules of Balb/C mice. There was a significant difference between the hyperthyroidism (experimental) and control groups. * P>0.05

**Figure 2:**
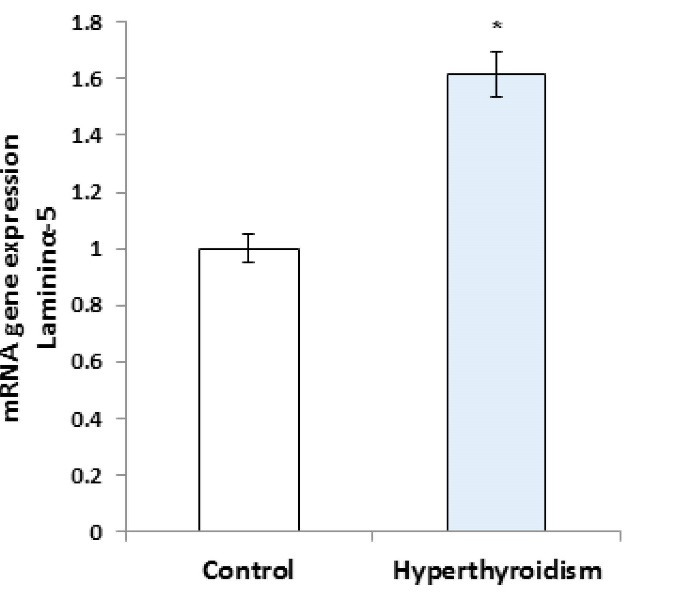
Collagen IV mRNA gene expression in the basement membrane of seminiferous tubules of Balb/C mice. There was no significant difference between the hyperthyroidism (experimental) and control groups. *P>0.05

**Figure 3:**
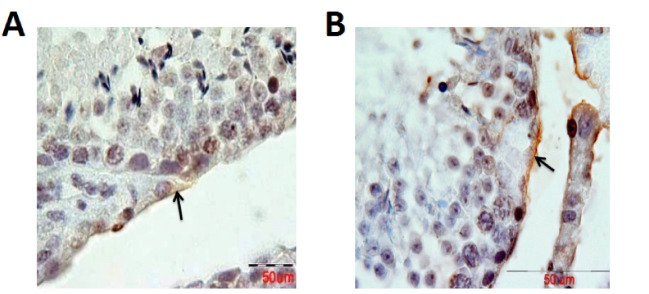
Transverse section from seminiferous tubule in hyperthyroidism (**A**) and control (**B**) groups. Immunohistochemistry method using antibodies against collagen IV counterstained with hematoxylin. Arrows are indicating the reaction of the anti-Collagen antibody with the collagen of beasmembrane and extracellular matrix. In the experimental group, the weak reaction contrary to the control group was observed (original magnification ×1000).

**Figure 4:**
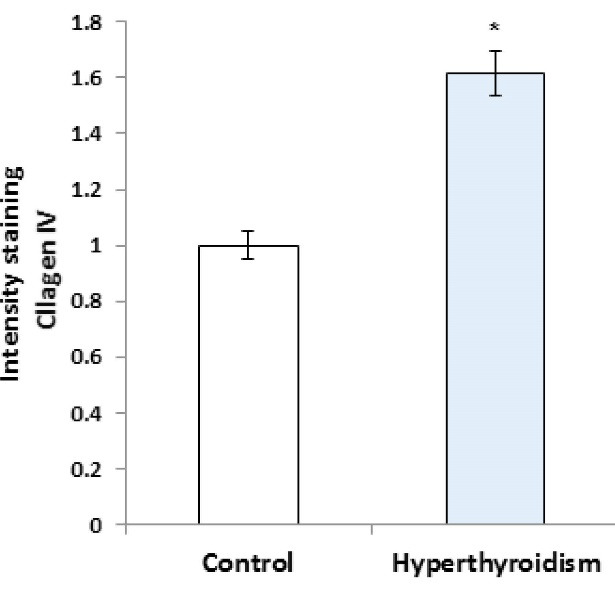
Collagen IV protein expression in the basement membrane of the seminiferous tubules of Balb/C mice. There was no significant difference between the hyperthyroidism (experimental) and control groups. *P>0.05

**Figure 5:**
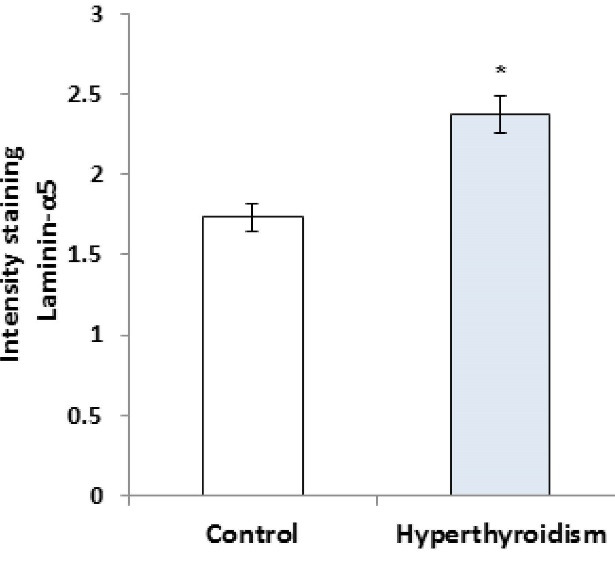
Laminin protein expression in the basement membrane of seminiferous tubules of Balb/C mice. There was a significant difference between the hyperthyroidism (experimental) and control groups. *P>0.05

**Figure 6:**
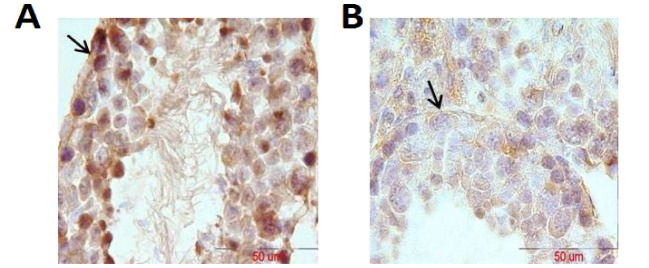
Transverse section through seminiferous tubule in hyperthyroidism (**A**) and control (**B**) groups. Immunohistochemistry method using antibodies against Laminin-α_5_ counterstained with hematoxylin. Arrows are indicating the reaction of the anti-Laminin antibody with the Laminin of beasmembrane and extracellular matrix. In the experimental group, a massive reaction in contrast control group was observed (original magnification ×1000).

RT-PCR results showed that the expression of laminin mRNA increased significantly in the experimental group in comparison with the control group (P=0.016, Figure-1), but the results of RT-PCR regarding the expression of collagen IV mRNA were not significantly different between the two groups (P>0.05, Figure-2). Besides, the reactions of the anti-collagen were very weak without any significant difference in seminiferous tubules BM between the hyperthyroidism group and the control group (P>0.05, Figures-3 and 4). According to the Mann-Whitney U test, immunohistochemical anti-laminin reactions on the seminiferous tubules BMs were increased significantly in the hyperthyroidism group in comparison with the control group (P=0.46, Figures-5 and 6). The classifications of immunoreactivity and intensity of reaction for laminin were weak (+) and moderate (++) in the control group, while they were strong (+++) and very strong (++++) in the hyperthyroidism (Table-1).

## Discussion

In this study, our findings showed that hyperthyroidism did not significantly affect collagen IV protein and gene expression. On the contrary, hyperthyroidism causes up-regulation of laminin protein and gene expression. With little discrepancy, most previous studies revealed the same findings. For example, Ulisse *et al*. [18] studied laminin and collagen IV in the BM of Sertoli cells in Wistar rats treated with T3. They observed that anti-laminin effects were increased in their experimental group compared to controls. In contrast, anti-collagen effects were decreased in the experimental group compared to controls. Further, Western Blot analysis corroborated these results [18]. Similarly, in our study, the same results were observed in seminiferous tubules BM of the hyperthyroidism group. In a study by Amini *et al*. on the effects of oral administration of sodium nitrite (with a concentration of 3 mg/L) on laminin expression in mice, it was found that immunochemical anti-laminin reaction was not significantly different between experimental and control groups [19]. Aldaghi *et al*. exhibited that laminin reaction in normal sciatic nerves was mainly observed in the perineurium, near the Schwann cells, and in the epi- and endoneurial blood vessels’ walls. Laminin in diabetic sciatic nerves was specifically in the inner layers of peri- and endoneurium, which immediately surrounds Schwann cells and the thickened layers of the epi- and endoneurial blood vessels’ walls. Treatment with insulin significantly reduced laminin expression in all these sites. However, treatment with alpha lipoic acid (ALA) significantly reduced the expression of laminin only in the epi- and endoneurial blood vessels’ walls. Data analyses about laminin mRNA gene expression was significantly reduced in diabetic groups compared to controls, but these results were not significant regarding ALA treatment [20]. Also, with regard to normal sciatic nerves, collagen IV was observed in the peri- and endoneurium near the Schwann cells and the BM of epi- and endoneurial blood vessels. In diabetic sciatic nerves, collagen IV was prominently observed in the perineurium and BM near the epi- and endoneurial blood vessels. In the study by Aldaghi *et al*. [21], the reaction of collagen IV in diabetic+insulin group reduced significantly in comparison with the diabetic group. In diabetic+ALA group, collagen IV reaction significantly decreased only in the epi- and endoneurial blood vessels in comparison with diabetic group. Further, RT-PCR showed collagen IV mRNA gene expression in sciatic nerves significantly increased in a diabetic group relative to a control group [21]. Planska *et al*. in a study on melanoma concluded that the expression of collagen IV decreased, but the expression of laminin increased in BM [22], which is similar to our findings. Mohammadi *et al*. in a study using a mouse model showed that nicotine-induced immunohistochemical reaction of collagen IV in the alveolar BM of the control group changed from light brown to dark brown [23], and in a similar investigation on pulmonary alveoli, laminin expression significantly decreased in experimental groups compared to control groups [24]. Effects of nitrate in drinking water on laminin-α5 expression in rat renal glomeruli showed that by increasing the concentration of nitrate in drinking water of Wistar rats, the severity of the reaction of anti-laminin-α5 decreased in renal glomeruli [25]. Also, there was no significant difference in lamininα_5_ expression among the group receiving water containing 45mg/L of nitrate compared to distilled water and control groups. However, they observed a significant increase in anti-lamininα_5_ reaction in a group treated with water containing 100mg/L of nitrate compared to another group [25]. These results were not in line with our findings regarding the effects of hyperthyroidism on lamininα_5_ gene expression. Loveland *et al*. [26] ascribed that collagen IV is detected in the extracellular matrix, the interstitial tissue, and seminiferous tubules BM on the 5th postnatal day in rats, which gradually increased from day 10 to day 20 in seminiferous tubules BM through immunostaining. In rats treated with propylthiouracil, collagen IV formation was delayed from day 10 to day 20. Also, high levels of collagen IV remained in the testicular extracellular matrix of rats treated with propylthiouracil [26]. These results were also contradictory with our findings regarding the effects of hyperthyroidism.

## Limitations of the Study

Blood collection from mice was challenging due to their low blood volume; thus, we used gel tubes provided by the laboratory and performed the required evaluations.

## Conclusion

Regarding the effects of hyperthyroidism on collagen IV and lamininα_5_, we obtained relatively similar findings to other diseases. Most studies addressing the effects of different substances or compounds on collagen and laminin such as our study approved that laminin immunohistochemical reactions increased in hyperthyroidism groups in comparison with control groups, but on the contrary, collagen IV immunohistochemical reactions decreased in hyperthyroidism groups in relative to control groups. However, in a few studies, these conclusions regarding laminin and collagen IV were not consistent with our results.

## Acknowledgment

I would like to thank all the colleagues and assistances who helped us in conducting the present study.

## Conflict of Interest

The authors declare that there is no conflict of interests associated with the publication of this paper.

**Table 1:** The Immunoreactivity Classification and Reaction Intensity for Collagen IV and Laminin-α5 Antibodies

**Reaction**	**Grade**
**Negative (-)**	0
**Weak (+)**	1
**Moderate (++)**	2
**Strong (+++)**	3
**Very strong (++++)**	4

(-), no reaction; (+), weak reaction: light brown; (++), moderate reaction: brown; (+++), strong reaction: dark brown and (++++), very strong reaction: very dark brown

**Table 2:** Sequences of Primers Used in this Study

**Genes**	**Primer Sequence (5’→3’)**
Laminin-α5	Forward	ATTCCTTTGTGATGCACACCAG
	Reverse	AAGCTGTAAGCATTCGCGTAGTA
Collagen IV	Forward	CGTCCCACAGGAATAGGCT
	Reverse	TACCAACGAAGGGCTGCG
GAPDH	Forward	AACTCCCATTCTTCCACCTTTG
	Reverse	CTGTAGCCATATTCATTGTCATACCA

**GAPDH:**Glyceraldehyde-3-phosphate dehydrogenase
